# Perceptions of Stigma Among Patients With Hepatitis B in Germany: Cross-Sectional Survey

**DOI:** 10.2196/66379

**Published:** 2025-06-13

**Authors:** Bettina M Zimmermann, Theresa Willem, Michael Rost, Nina Matthes, Alena Buyx

**Affiliations:** 1 Institute of History and Ethics in Medicine, TUM School of Medicine and Health, Technical University of Munich, Ismaninger Str. 22, Munich, 81675, Germany, 49 8941404041; 2 Institute for Molecular Immunology, TUM School of Medicine and Health, Klinikum Rechts der Isar, Technical University of Munich, Munich, Germany; 3 Institute of Philosophy, Multidisciplinary Center for Infectious Diseases, University of Bern, Bern, Switzerland; 4 Department of Science, Technology and Society (STS), School of Social Sciences and Technology, Technical University of Munich, Munich, Germany; 5 Institute for Biomedical Ethics, University of Basel, Basel, Switzerland

**Keywords:** infectious diseases, infection, contaminate, septic shock, hepatitis B, social stigma, stigmatize, discrimination, prejudice, social factor, social disparity, social inequality, social inequity, Germany

## Abstract

**Background:**

Many studies find associations between hepatitis B and stigma, but studies from the Western European context are lacking. Based on available studies, we hypothesized that younger age, higher education, male gender, higher privacy needs, and non-German mother tongue were positively associated with perceived hepatitis B–related stigma.

**Objective:**

This study aims to describe the prevalence of perceived social stigma among patients with hepatitis B in Germany and to assess what factors are associated with perceptions of hepatitis B–related stigma.

**Methods:**

Applying the short version of the Berger stigma scale, we surveyed 195 patients with hepatitis B about their perceptions of hepatitis B–related stigma, privacy needs, and demographic variables through a paper-based questionnaire. Venue-based recruitment of adult patients diagnosed with acute or chronic hepatitis B was implemented at 3 clinical centers in Germany. Patients who could not read German were excluded from the study.

**Results:**

From the 195 valid questionnaires, 45.1% (88/195) of participants identified as female, 36.6% (71/195) had a high school diploma, and 56.9% (111/195) reported a mother tongue other than German. The mean (SD) stigma score throughout the sample was 5.52 (6.02; range 0-24) and the median was 3.50 (IQR=9.75). Regression analysis revealed that non-German mother tongue, individual data privacy needs, and participants’ secrecy regarding their hepatitis B diagnosis independently predicted perceived hepatitis B-related stigma. More precisely, the higher the data privacy need and the more secret the hepatitis B diagnosis, the higher the perceived stigma, and perceived stigma was higher for patients with a non-German mother tongue. Age, gender, and education were no predictors of perceived stigma.

**Conclusions:**

The surveyed patients with hepatitis B in Germany reported lower levels of hepatitis B–related stigma than found in other studies conducted in Asian countries. The association with non-German mother tongue indicates an important cultural and social component in the perception of stigma. Community-based interventions and the sensibilization of health care professionals might help overcome perceptions of stigma among hepatitis B–affected populations.

## Introduction

### Background

Viral hepatitis encompasses a spectrum of liver diseases resulting from hepatotropic viral infections affecting hepatocytes. The 5 hepatotropic viruses, hepatitis types A, B, C, D, and E, exhibit varied acute manifestations, with outcomes ranging from spontaneous resolution to chronic infection [1]. The hepatitis B virus (HBV) is primarily transmitted through infected blood or body fluids, including unprotected sexual intercourse, needle-sharing, or perinatal exposure [2]. Chronic HBV infections affect over 3% of the world’s population and pose substantial health risks, including cirrhosis and liver cancer [3]. While Germany is categorized as a low-prevalence country for HBV, with a prevalence of 0.3% in the general population [4], a disproportionately high burden of HBV exists among immigrants from high-endemic countries who are estimated to contribute 49% of chronic HBV cases in Germany [5]. Although prophylactic vaccinations against HBV are available, the absence of a cure for chronic infections underscores the importance of ethical considerations in clinical research involving patients affected by HBV infections [6].

One particularly relevant ethical and social aspect in the context of infectious diseases, such as hepatitis B, is stigma [7,8]. Stigma is a “social process, experienced or anticipated, characterized by exclusion, rejection, blame or devaluation” [7] where “some characteristic or behavior of others [are deemed] as falling short of communal norms of desirability” [9]. Stigma can be social (leading, for instance, to social isolation or loss of social status), internalized (referring to a person’s perception or anticipation of being socially rejected), or structural (causing disadvantages in accessing high-quality social goods, such as access to health care or university admissions) [10]. People affected by infectious diseases (especially chronic forms) are prone to stigma because of the transmissibility of the disease: the fear of infecting others might lead to social isolation and internalized stigma [11]. However, even uninfected individuals who share characteristics associated with this disease tend to be stigmatized. For example, men who have sex with men and sex workers tend to be associated with sexually transmitted infections (ie, from HIV or HBV) and may suffer from the same stigma even if unaffected [12]. Stigma has been shown to negatively affect the quality of life [13] and mental health [14] of people with hepatitis B. Hepatitis B–related stigma also leads to reduced screening rates in at-risk populations [15,16], lower adherence to antiviral treatment [17] and presents a barrier to accessing health care [18]. Thus, stigma is a major barrier in hepatitis B treatment, prevention, and elimination [19,20].

Against this backdrop, many studies worldwide have assessed stigma related to infectious diseases. A study from Hong Kong reported that around one-third of HIV-affected participants experienced discrimination due to their condition, particularly by health care professionals [21]. Patients affected by chronic hepatitis C also experience social and internalized stigma [22], and some comparative studies report higher stigma scores among patients with hepatitis C as compared with hepatitis B [8,23]. Still, studies report considerable ratios of stigma among patients with hepatitis B, ranging from high perceptions of stigma in 47.4% of surveyed patients in Turkey [23] to over 60% in India [10]. Another Turkish survey found 19.5% of hepatitis B-affected participants reporting experienced stigma, and 27.4% were worried about stigma [24]. Toumi et al [11] illustrate self-stigma and report on experienced social stigma around people affected by hepatitis B in several world regions. Overall, hepatitis B–related stigma was reported to be insufficiently described in populations outside Asia [25].

### Study Hypotheses

This study addresses this gap by reporting perceptions of stigma among patients with hepatitis B in Germany. It also aims to identify factors associated with perceptions of hepatitis B–related stigma. To this end, we developed the following hypotheses based on findings in other studies (referenced below) investigating factors associated with stigma related to hepatitis B or C:

H1: Men with hepatitis B report higher stigma than women with hepatitis B [23,26].

H2: Patients with hepatitis B with higher general education (high school degree) report higher stigma [27].

H3: Older age is associated with less perception of stigma [28].

H4: Patients with hepatitis B and a mother tongue other than German report higher stigma than those with solely a German mother tongue [29].

Further, qualitative interviews with patients with hepatitis B (results are reported elsewhere [30]) suggest a strong connection between privacy attitudes and stigma. Since we measured privacy attitudes in terms of the secrecy of HBV diagnosis and general data privacy needs, we further hypothesize:

H5a: Higher perceptions of stigma are associated with higher secrecy regarding HBV diagnosis.

H5b: Higher perceptions of stigma are associated with higher data privacy needs.

## Methods

### Overview

This study is part of the European Union–funded international research consortium “TherVacB–A Therapeutic Vaccine to Cure hepatitis B.” As part of work package 6, our research team aimed to assess the ethical, legal, and social aspects of social media recruitment. Preceding the here-presented survey study, a qualitative multistakeholder interview study was conducted and informed survey construction. The primary aim of the subsequent quantitative survey study among patients with hepatitis B in Germany was to assess the acceptance of social media recruitment. We report the findings of this original survey study elsewhere [30]. This paper presents a secondary analysis from this survey study based on the same questionnaire, investigating patients with hepatitis B perceptions of stigma and factors associated with stigma perceptions. Reporting follows the STROBE (Strengthening the Reporting of Observational Studies in Epidemiology) guidelines [31].

Study nurses distributed a paper-based questionnaire to adults diagnosed with acute or chronic hepatitis B through 3 clinical centres in Germany (Munich, Hannover, and Leipzig). Even though study nurses were instructed to hand out the questionnaire to every incoming patient with hepatitis B (hospitals estimated n=939) and to avoid self-selection, only 285 questionnaires (22%) were distributed. The main reason was an overburden of workload from the study nurses in the aftermath of the COVID-19 pandemic. Therefore, the data collection period was extended from initially 7 to a total of 12 months (June 2022 to May 2023) until the goal of collecting 200 questionnaires was reached. The required sample size was calculated using G*Power, a free tool to compute statistical power analyses developed by researchers from the Heinrich Heine University Dusseldorf [32]. Given an effect size Cohen f_2_ of 0.15, an α error probability of .05, and 11 predictors, 178 participants would result in a power of 0.95. Data from the paper-based questionnaires were typed manually into an excel sheet. Data entry was double checked by a second researcher for correctness.

### Ethical Considerations

The ethics committees from the Technical University of Munich (12/22-S-NP), Hannover Medical School (10368_BO_K_2022), and University Clinic Leipzig (189/22-lk) approved the study. Informed consent was obtained from all participants in this study. To protect their privacy, participants gave their consent by ticking a box at the beginning of the questionnaire rather than signing a consent form. For opting out, participants had to keep and state a unique identifier that they received on their study information leaflet. No names or other identifying information was collected. Participants did not receive any compensation for participating in this study.

### Survey Construction

Survey construction involved several steps. First, based on the research team’s expertise and findings of a preceding qualitative study, we discussed potential independent variables that possibly predict perceived stigma (see the hypotheses mentioned above). Then, we identified validated questionnaires that captured possible predictors or the dependent variable. If no validated tools existed for a variable of interest, we developed scales for the respective variables. The compiled survey was pretested with 6 patients with hepatitis B (3 of them with a non-German mother tongue), leading to minor changes.

The survey included questions about (1) participants’ general use of social media, (2) self-reported digital literacy, (3) participants’ use of social media in connection to their hepatitis B infection, (4) their interest in clinical studies, (5) trust in medical and non-medical information sources, (6) acceptance of social media as a recruitment tool for clinical studies, (7a) their attitudes regarding data privacy, (7b) perceived secrecy of their hepatitis B, and (8) perceived stigma related to hepatitis B. Because this is a secondary study, we included only variables with a theoretical or empirical connection to stigma in this analysis, excluding variables about social media (no. 1, 2, 3, 6), interest in clinical studies (no. 4) and trusted information sources (no. 5). Responses were measured with a 5-point Likert scale from 0=never or do not agree at all, to 4=always or fully agree. The full questionnaire is available in Multimedia Appendix 1.

Perceived hepatitis B–related stigma was measured using a 6-item version of the hepatitis C stigma scale [33]. This scale was modified from the validated Berger HIV stigma scale [34], and the short 6-item version showed comparable results to the original 40-item scale [33]. For our questionnaire, “hepatitis C” was replaced by “hepatitis B,” and the original items were translated into German by back-and-forth translation by a professional translator. This scale showed excellent reliability in our sample (*α*=.931). Secrecy and data privacy needs were self-developed and included 2 items each. For secrecy, measuring the willingness of patients with hepatitis B to tell people they know about their condition, the items were “*My family and friends know about my Hepatitis B infection*” and “*My hepatitis B infection is a secret*” (Multimedia Appendix 1 ). For data privacy, the items were “*I am careful not to disclose anything about my hepatitis B infection on social media for fear that the platform will collect and store this information*” and “*I want my medical data in connection with my illness to be particularly well protected*.” Both scales were developed based on findings with qualitative interviews including 6 patients with hepatitis B [30] and showed acceptable reliability (secrecy: *α*=.623; data privacy needs: α=.587). Besides these adapted and self-developed scales, we included 4 demographic variables in the analysis (age, gender, education, and mother tongue).

### Statistical Analysis and Predictor Selection

We used SPSS (version 28.0 IBM) for data analysis, performing descriptive analyses and determining independent factors associated with participants’ perceived stigma using multiple linear regression analysis. The statistical significance level was set at *P*<.05. For multiple linear regression analysis, assumption checks were performed before the interpretation of the model (Multimedia Appendix 2).

For our analysis, a literature review and hypotheses derived from our previous qualitative study determined predictor selection (hypotheses above). In addition, the sample-size and predictor ratio a priori determines variable selection for regression modeling. According to Harrell, a fitted regression model is likely to be reliable when *P*<*m*/10 or *P*<*m*/20 (average requirement: *P*<*m*/15), where *P* is the number of predictors and *m* is the sample size [35]. Applying this requirement to our sample size (N=195) and having missing data (80.6% of variables, 28.7% of cases, and 3.7% of values), the *a priori* limitation was 11 included predictors. The presented analysis included 6 predictors in the regression model for which we had hypothesized an association with hepatitis B–related stigma: age, gender (dichotomous), education (dichotomous), German or non-German mother tongue (dichotomous), secrecy of HBV diagnosis, and data privacy needs related to HBV diagnosis.

## Results

### Sociodemographic Characteristics of the Sample

From the 285 questionnaires distributed, 207 (72.6%) were returned. Because 12 questionnaires had to be excluded due to lack of consent, the final analysis is based on 195 questionnaires. Table 1 portrays the participants’ demographic characteristics.

**Table 1.** Participant characteristics (N=195).

Characteristics	Values, n (%)	
Gender		
Men	101 (51.8)	
Women	88 (45.1)	
Missing	6 (3.1)	
Age (years)		
18‐29	16 (8.2)	
30‐39	50 (25.6)	
40‐49	58 (29.7)	
50‐59	38 (19.5)	
More than 60	24 (12.3)	
Missing	9 (4.6)	
Education: highschool diploma		
Yes	71 (36.4)	
No	110 (56.4)	
Missing	14 (7.2)	
Mother tongue (multiple answers possible**)**		
German	101 (51.8)	
Other	111 (56.9)	
Missing	12 (6.2)	
Total	195 (100)	

### Description of Scales

The perceived hepatitis B–related stigma score was calculated by building the sum of the 6 items (range 0-24). We excluded 15/195 (7.7%) questionnaires because, in these, not all items were answered. The mean score throughout the sample was 5.52 (SD 6.02), and the median was 3.50 (IQR 9.75. As Table 2 indicates as many as 50/180 (27.8%) participants who completed this score indicated zero perceived hepatitis B–related stigma, selecting “completely disagree” to all 6 items that asked them about various dimensions of hepatitis B–related stigma (Table 2).

**Table 2.** Distribution of perceived stigma score (N=180). The summative stigma score was calculated by building the sum of the 6 stigma-related items.

Summative stigma score	Case count
0	50
1	18
2	8
3	11
4	9
5	11
6	10
7	8
8	3
9	2
10	4
11	9
12	10
13	6
14	5
15	3
16	2
17	1
18	3
19	1
20	2
21	1
22	1
23	0
24	3

### Regression Analysis

**Figure 1.**
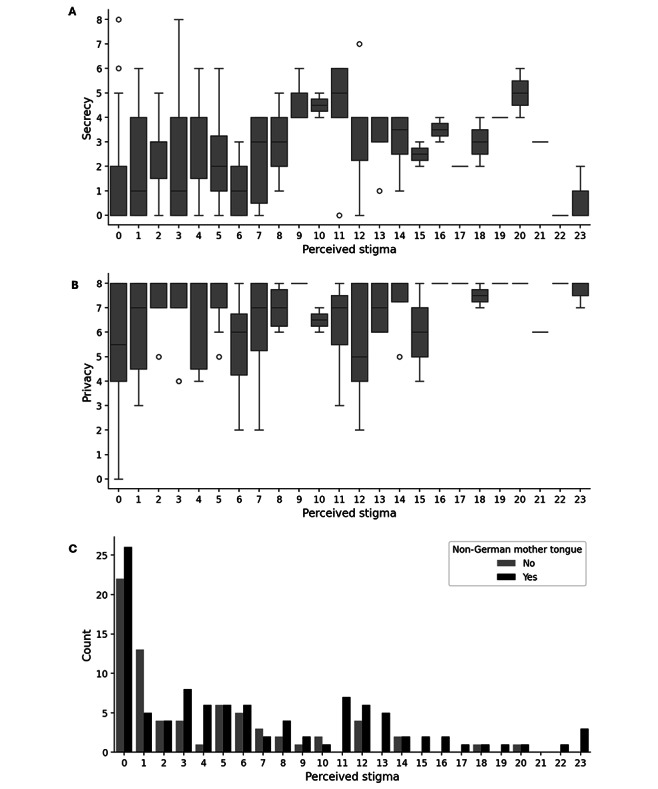
Relationships between perceived stigma and (**A**) secrecy, (**B**) privacy, and (**C**) non-German mother tongue.

Using multiple linear regression analyses, we evaluated predictors’ associations with participants’ perceived hepatitis B–related stigma. Testing the statistical significance of the overall model fit, the *F* test indicated that the predictors included in the model significantly contributed to the explanation of perceived hepatitis B–related stigma as the dependent variable (Table 3). Regression analysis revealed that a non-German mother tongue, privacy, and the secrecy of the HBV diagnosis independently predicted perceived stigma. More precisely, the higher the data privacy needs and the more secret hepatitis B, the higher the perceived stigma, and perceived stigma was higher for patients with non-German mother tongue (Table 3, Figure 1).

**Table 3.** Multiple linear regression analysis (n=161, dependent variable: perceived hepatitis B–related stigma)^a^.

	Unstandardized coefficients (B)	SE	β	*t* test (*df*)	*P* value	Tolerance	Variance inflation factor
Constant	−2.700	2.978	N/A^c^	−.907 (154)	.37	N/A^c^
Age	.025	.040	.049	.623 (154)	.53	.882	1.134
Gender^b^	−.255	.893	−.021	−.286 (154)	.78	.972	1.029
Education^b^	−.321	.914	−.026	−.351 (154)	.72	.948	1.054
Non-German mother tongue^b^	3.318	.941	.272	3.525 (154)	<.001	.903	1.107
Secrecy	.522	.224	.182	2.333 (154)	.02	.882	1.134
Data privacy needs	.715	.236	.239	3.034 (154)	.003	.864	1.157

a Overall model fit: *F*_6154_=5.293, *P*<.001; *R*2=.171; n=161

b Dichotomous items.

c N/A = not applicable

## Discussion

### Principal Results

The majority of study participants (patients with hepatitis B living in Germany) report moderate levels of perceived stigma. In their systematic review, Smith-Palmer et al [25] (2020) concluded that hepatitis B–related stigma was poorly characterized in non-Asian populations. Our findings suggest that perceived stigma might be lower among German-speaking patients with hepatitis B in Germany than among the other populations studied, where strong experiences or perceptions of stigma ranged from 20%‐60% of surveyed patients with hepatitis B [10,23,24]. However, quantitative comparisons between studies are difficult because stigma is measured in different ways in the scholarly literature. Comparative studies accounting for cultural dimensions of stigma and potential language bias have to confirm whether perceived stigma is lower in Germany than in Asian populations.

### The Role of Mother Tongue

Despite the above-mentioned under-representation of patients with hepatitis B with insufficient German language skills, the sample was large and diverse enough to show significantly higher perceptions of stigma in participants with a mother tongue other than German (confirming H4, Table 4). A study by Dam et al [36] juxtaposed stigma perception in Vietnamese patients with hepatitis B in Vietnam and Vietnamese Americans in the United States and found considerable differences in stigma perceptions, with the cohort in Vietnam being more progressive in their perspectives about hepatitis B and its attached stigma than the American immigrant cohort. This might represent an additional explanation for the relatively low overall levels of stigma measured in our cohort as compared with Asian cohorts.

Yet, in our study, patients with a non-German mother tongue perceived more hepatitis B–related stigma than people with a German mother tongue. Even though it was not possible in our study to analyze where participants with non-German mother tongue originated from, Dam et al [36] study pertains to the broader social context of stigma, indicating that immigration decontextualizes individuals from their social environments, which might change how they expect their environment to perceive them. Since participants with non-German mother tongue (or their families) most likely have an immigration background, our study speaks to the confounding impacts of social context changes due to immigration on immigrant patients’ perceptions of illness and its attached stigma [37]. Indeed, a qualitative study among Chinese immigrants in Australia found that hepatitis B–related stigma mainly manifested in anticipated stigma and rooted in the ethnic and cultural backgrounds of the community [38]. Further studies are needed to assess perceptions of disease-related stigma in other populations of foreign origin.

### Privacy Needs and Vulnerability

Patients reporting higher hepatitis B–related stigma also reported higher data privacy needs and more strongly perceived their diagnosis as a secret, confirming H5a and H5b (Table 4). This confirms findings from a qualitative interview study of our group that included 6 patients with hepatitis B patients from Germany [30,39]. Within this interview study, a predominant concern voiced by the interviewed patients revolved around the potential compromise of their privacy. Patients were worried that the information they shared on social media platforms could be easily disseminated or reposted without their consent. Interviewed patients expressed apprehension regarding the possible unauthorized dissemination or reposting of their health data on social media platforms, highlighting their fear of potential breaches of confidentiality. Their apprehension stems from the anticipated societal judgment and discrimination that could arise upon the disclosure of their HBV diagnosis.

Stigma (whether anticipated or experienced) adds an important layer of vulnerability to patients [40]. Thus, data privacy should be particularly strict for patients with stigmatized conditions. This affects, in particular, public outreach programs and online community work, for instance, on social media [39]. Hepatitis B–related health complications, as well as immigration status that comes with challenges related to poor language skills, assimilation, and cultural imprinting, may add additional layers of vulnerability, leading to cascading effects that exacerbate vulnerability [41]. Privacy-preserving public health programs targeted toward high-prevalence populations that aim to inform and prevent infection should also address these perceptions of stigma and the exacerbating vulnerabilities within those populations. Such community-based interventions and the sensibilization of health care professionals might help overcome remaining perceptions of stigma among hepatitis B–affected populations. They might contribute to increased testing rates in at-risk populations [42].

Contrary to what we had hypothesized, gender (H1), age (H3), and education (H2) were no predictors of stigma (Table 4). However, disease-related literacy, which is what is portrayed in the literature to be negatively associated with stigma [10], was not measured directly. As suggested in other studies 17], it might still be relevant that sociomedical education regarding local perceptions of stigma (particularly in countries with low stigma rates) could serve as a harm-mitigating factor in highly stigmatized patient communities.

**Table 4.** Outcomes of study hypotheses.

Hypotheses	Outcome
(H1) Men with hepatitis B report higher stigma than women with hepatitis B [23,26].	Rejected
(H2) Patients with hepatitis B with higher general education (high school degree) report higher stigma [27].	Rejected
(H3) Older age is associated with less perception of stigma [28].	Rejected
(H4) Patients with hepatitis B with a mother tongue other than German report higher stigma than those with a German mother tongue [29].	Confirmed
(H5a) Higher perceptions of stigma are associated with higher secrecy regarding HBV^a^ diagnosis.	Confirmed
(H5b) Higher perceptions of stigma are associated with higher data privacy needs.	Confirmed

a HBV: hepatitis B virus.

### Limitations

Even though the survey sample is representative of the hepatitis B population regarding age and gender when compared with the most recently measured prevalence of chronic hepatitis B in Germany [4], a relevant selection bias might lead to an underestimation of perceived stigma among patients with hepatitis B in Germany. First, the venue-based recruitment in 3 major clinical centres might have contributed to such a selection bias. Second, the questionnaire was only available in the German language, which led to an underrepresentation of patients who have insufficient German language skills to understand the survey. The questionnaire was also not designed with respect to cultural aspects of stigma from different immigrant populations.

Representing an additional study limitation, this paper reports a secondary analysis and stigma was not the primary dependent variable when the survey was designed [30]. Therefore, some variables that are potentially associated with stigma were not included in this analysis but might have increased the explanatory power of the regression model. Such variables that have been shown in other studies to affect perceptions of stigma are disease-related knowledge [10,43,44], patients’ socioeconomic status or employment level [10], comorbidities [28], or family history of hepatitis B [27]. Other potentially relevant factors that were not measured include occupation, geographic location (urban or rural), duration of HBV diagnosis, or access to social support. Our regression model explained 17% of the variance of stigma perception, pointing to the multifaceted nature of stigma perceptions. We also used the short version of the stigma scale to increase the chance that patients would complete the survey and did not distinguish between perceived, enacted, and internalized stigma. While this short version showed excellent reliability and was validated [34], no in-depth analysis of stigma subscales was possible.

Because the original study focused on social media, there are potential framing effects that participants might target their answers to social media contexts. However, the acceptance of social media as a recruitment tool for hepatitis B–related clinical studies was low in this cohort and secrecy levels were high [30]. Since we would expect framing effects to increase stigma perceptions in this context, the findings of low stigma perceptions seem robust.

### Conclusions

This is the first study in a Western European setting assessing perceived stigma among patients with hepatitis B. Despite its limitations related to the secondary analysis of this survey, the study adds to the evidence of perceived hepatitis B–related stigma, which has been predominantly conducted in Asian settings. The survey revealed that self-reported hepatitis B-related stigma was less pronounced among German-speaking patients with hepatitis B in Germany than reported in studies conducted in other world regions. Yet, participants with a non-German mother tongue perceived more hepatitis B–related stigma than those with a German mother tongue. While this study excludes patients with hepatitis B who do not speak German, these findings point to the need for further research into the context of stigma perceptions among patients with immigrant backgrounds in Germany and other understudied European contexts. High perceptions of stigma were also associated with higher secrecy around the HBV diagnosis and higher data privacy needs, indicating the need to use privacy-preserving strategies in hepatitis B–related public health programs.

## Supplementary material

10.2196/66379 Multimedia Appendix 1 Questionnaire.

10.2196/66379 Multimedia Appendix 2 Supplementary methods.

## Abbreviations

HBV: hepatitis B virus
STROBE: Strengthening the Reporting of Observational studies in Epidemiology
