# Supplementary material for: Perceptions of Stigma Among Patients With Hepatitis B in Germany: Cross-Sectional Survey
Source: JMIR Form Res. 2025 Jun 13;9:e66379. doi: 10.2196/66379 (PMC12180674; doi:10.2196/66379)
Supplement: Multimedia Appendix 1 [file formative-v9-e66379-s001.docx]

# **Questionnaire**

Dear patient

Thank you very much for taking part in this study. Your participation is very valuable to us. We are contacting you because you suffer from hepatitis B. In this study, we are investigating how hepatitis B patients perceive new forms of recruitment for clinical trials. Please read the enclosed study information carefully before completing this questionnaire. If you have any questions, please contact the clinic staff or the study coordinator: [contact information redacted]

**🞏 I have read the information and agree to the processing of my data**

*Please note: Without this tick we are not allowed to use your questionnaire for the study and are obliged under data protection law to destroy it.*

Thank you for your consent. It will take about 20 minutes to complete the questionnaire. Please read everything carefully before you answer. Please complete the questionnaire as fully as possible, as this is very important for the validity of the study.

Social media are digital platforms for sharing information and life content. They are also sometimes used to recruit participants for scientific studies. First of all, we would like to know which social media you use and how often. How often do you use the following social media?

|  | | Never | Once a month or less | 2-4 times a month | Several times a week | Several times a day |
| --- | --- | --- | --- | --- | --- | --- |
| 1.01 | WhatsApp | 🞏 | 🞏 | 🞏 | 🞏 | 🞏 |
| 1.02 | Telegram | 🞏 | 🞏 | 🞏 | 🞏 | 🞏 |
| 1.03 | Youtube | 🞏 | 🞏 | 🞏 | 🞏 | 🞏 |
| 1.04 | Facebook | 🞏 | 🞏 | 🞏 | 🞏 | 🞏 |
| 1.05 | Instagram | 🞏 | 🞏 | 🞏 | 🞏 | 🞏 |
| 1.06 | Pinterest | 🞏 | 🞏 | 🞏 | 🞏 | 🞏 |
| 1.07 | Twitter | 🞏 | 🞏 | 🞏 | 🞏 | 🞏 |
| 1.08 | Xing | 🞏 | 🞏 | 🞏 | 🞏 | 🞏 |
| 1.09 | LinkedIn | 🞏 | 🞏 | 🞏 | 🞏 | 🞏 |
| 1.10 | Snapchat | 🞏 | 🞏 | 🞏 | 🞏 | 🞏 |
| 1.11 | Reddit | 🞏 | 🞏 | 🞏 | 🞏 | 🞏 |
| 1.12 | TikTok | 🞏 | 🞏 | 🞏 | 🞏 | 🞏 |
| 1. 13 | Tumblr | 🞏 | 🞏 | 🞏 | 🞏 | 🞏 |

Next, we would like to know how familiar you are with social media. How strongly would you agree with the following statements?

Seite 1

|  | | Do not agree at all | Rather disagree | Partly agree | Tend to agree | Fully agree |
| --- | --- | --- | --- | --- | --- | --- |
| 2.01 | I know how to create a social media account. | 🞏 | 🞏 | 🞏 | 🞏 | 🞏 |
| 2.02 | I know how to delete my social media account. | 🞏 | 🞏 | 🞏 | 🞏 | 🞏 |
| 2.03 | I know how to deactivate my social media account. | 🞏 | 🞏 | 🞏 | 🞏 | 🞏 |
| 2.04 | I know how to share content such as fotos in my social media account. | 🞏 | 🞏 | 🞏 | 🞏 | 🞏 |
| 2.05 | I know how to remove content from my social media account. | 🞏 | 🞏 | 🞏 | 🞏 | 🞏 |
| 2.06 | I know the copyright laws relevant to social media. | 🞏 | 🞏 | 🞏 | 🞏 | 🞏 |
| 2.07 | I know how to meet conflicts on social media | 🞏 | 🞏 | 🞏 | 🞏 | 🞏 |
| 2.08 | I know the social media guidelines in my professional activities. | 🞏 | 🞏 | 🞏 | 🞏 | 🞏 |
| 2.09 | I know how to verify the truthfulness of information shared on social media. | 🞏 | 🞏 | 🞏 | 🞏 | 🞏 |
| 2.10 | I know how to use different information sources to verify information from social media. | 🞏 | 🞏 | 🞏 | 🞏 | 🞏 |
| 2.11 | I can assess whether information on social media is true or false | 🞏 | 🞏 | 🞏 | 🞏 | 🞏 |
| 2.12 | Platforms like Facebook control what I see on social media. | 🞏 | 🞏 | 🞏 | 🞏 | 🞏 |
| 2.13 | Information I post on social media is permanent. | 🞏 | 🞏 | 🞏 | 🞏 | 🞏 |
| 2.14 | The ads I see on social media are specifically targeted to my preferences. | 🞏 | 🞏 | 🞏 | 🞏 | 🞏 |

So far, the focus has been on how you use social media in general. The following section will now focus on the use of social media in relation to your hepatitis B disease. How often do you use the following social media in relation to your hepatitis B disease? If you do not use social media, please indicate which areas of application you could imagine in principle.

|  | | Never | Rarely | Occasionally | Frequently | Very frequently |
| --- | --- | --- | --- | --- | --- | --- |
| 3.01 | To exchange with other patients | 🞏 | 🞏 | 🞏 | 🞏 | 🞏 |
| 3.02 | To find reliable medical information | 🞏 | 🞏 | 🞏 | 🞏 | 🞏 |
| 3.03 | To keep up with the latest research results | 🞏 | 🞏 | 🞏 | 🞏 | 🞏 |
| 3.04 | To learn about new clinical studies for new treatment options for Hepatitis B. | 🞏 | 🞏 | 🞏 | 🞏 | 🞏 |
| 3.05 | To get in touch with trustworthy researchers and study coordinators. | 🞏 | 🞏 | 🞏 | 🞏 | 🞏 |
| 3.06 | To find treating physicians. | 🞏 | 🞏 | 🞏 | 🞏 | 🞏 |

Clinical trials are scientific studies to research new medical treatments. This part of the questionnaire is about recruiting participants for hepatitis B clinical trials. The first question is how interested you are in participating in a clinical trial. How strongly would you agree with the following two statements?

|  | | Do not agree at all | Rather disagree | Partly agree | Tend to agree | Fully agree |
| --- | --- | --- | --- | --- | --- | --- |
| 4.01 | I am generally willing to participate in clinical trials | 🞏 | 🞏 | 🞏 | 🞏 | 🞏 |
| 4.02 | Participating in clinical trials for Hepatitis B is important to me. | 🞏 | 🞏 | 🞏 | 🞏 | 🞏 |

Seite 2

Now we are interested in how trustworthy you consider various sources to be when you first learn about a hepatitis B clinical trial. How trustworthy do you consider the information about a hepatitis B clinical trial to be when you receive it from this source?

|  | | Not at all | Rather not | Partly | Rather yes | Very |
| --- | --- | --- | --- | --- | --- | --- |
| 5.01 | Treating physician | 🞏 | 🞏 | 🞏 | 🞏 | 🞏 |
| 5.02 | Other medical professionals (nurses, assistants, administrative hospital staff, etc.) | 🞏 | 🞏 | 🞏 | 🞏 | 🞏 |
| 5.03 | Other patients | 🞏 | 🞏 | 🞏 | 🞏 | 🞏 |
| 5.04 | Public poster ads | 🞏 | 🞏 | 🞏 | 🞏 | 🞏 |
| 5.05 | Newspaper ads | 🞏 | 🞏 | 🞏 | 🞏 | 🞏 |
| 5.06 | TV ads | 🞏 | 🞏 | 🞏 | 🞏 | 🞏 |
| 5.07 | Online platforms (specialized clinical trial platforms) | 🞏 | 🞏 | 🞏 | 🞏 | 🞏 |
| 5.08 | Study-specific website | 🞏 | 🞏 | 🞏 | 🞏 | 🞏 |
| 5.09 | Social media ads | 🞏 | 🞏 | 🞏 | 🞏 | 🞏 |
| 5.10 | Social media: Personal message from an unknown source | 🞏 | 🞏 | 🞏 | 🞏 | 🞏 |
| 5.11 | Social media: Personal message from the study leader | 🞏 | 🞏 | 🞏 | 🞏 | 🞏 |

The next section is about your attitude towards social media for recruitment for hepatitis B clinical trials. How strongly would you agree with the following statements?

|  | | Do not agree at all | Rather disagree | Partly agree | Tend to agree | Fully agree |
| --- | --- | --- | --- | --- | --- | --- |
| 6.01 | Social media are well suited to make patients aware of studies on new hepatitis B treatments. | 🞏 | 🞏 | 🞏 | 🞏 | 🞏 |
| 6.02 | Social media increase the likelihood of success in hepatitis B clinical trials. | 🞏 | 🞏 | 🞏 | 🞏 | 🞏 |
| 6.03 | I would be recruited via social media for a hepatitis B clinical trial. | 🞏 | 🞏 | 🞏 | 🞏 | 🞏 |
| 6.04 | I would use social media to learn about hepatitis B clinical trials. | 🞏 | 🞏 | 🞏 | 🞏 | 🞏 |
| 6.05 | It is difficult for me to find appropriate channels to learn about hepatitis B clinical trials. | 🞏 | 🞏 | 🞏 | 🞏 | 🞏 |

You're almost there! This part of the survey is about your privacy concerns related to your hepatitis B disease. For each question, please answer how strongly it applies to you.

|  | | Do not agree at all | Rather disagree | Partly agree | Tend to agree | Fully agree |
| --- | --- | --- | --- | --- | --- | --- |
| 7.01 | My family and friends know about my Hepatitis B infection. | 🞏 | 🞏 | 🞏 | 🞏 | 🞏 |
| 7.02 | My hepatitis B infection is a secret. | 🞏 | 🞏 | 🞏 | 🞏 | 🞏 |
| 7.03 | I am careful not to disclose anything about my hepatitis B infection on social media for fear that the platform will collect and store this information. | 🞏 | 🞏 | 🞏 | 🞏 | 🞏 |
| 7.04 | I want my medical data in connection with my illness to be particularly well protected. | 🞏 | 🞏 | 🞏 | 🞏 | 🞏 |

Seite 3

We are also interested in the social circumstances of your hepatitis B disease. Some of the following questions assume that others know about your hepatitis B disease. This may not be the case for you. In this case, please imagine that you are in this situation. How strongly would you agree with the following statements?

|  | | Do not agree at all | Rather disagree | Partly agree | Tend to agree | Fully agree |
| --- | --- | --- | --- | --- | --- | --- |
| 8.01 | Other people's reactions to my hepatitis B infection hurt me. | 🞏 | 🞏 | 🞏 | 🞏 | 🞏 |
| 8.02 | Some people avoid touching me as soon as they find out about my my hepatitis B infection. | 🞏 | 🞏 | 🞏 | 🞏 | 🞏 |
| 8.03 | Some people don't want me around their children once they learn about my hepatitis B infection. | 🞏 | 🞏 | 🞏 | 🞏 | 🞏 |
| 8.04 | Other people physically backed away from me when they learned that I had hepatitis B. | 🞏 | 🞏 | 🞏 | 🞏 | 🞏 |
| 8.05 | I stopped seeing some people because of their reactions to my hepatitis B disease. | 🞏 | 🞏 | 🞏 | 🞏 | 🞏 |
| 8.06 | People seem to be afraid of me as soon as they find out about my hepatitis B infection. | 🞏 | 🞏 | 🞏 | 🞏 | 🞏 |

Finally, we would like to ask you for some personal details. This is very important for the evaluation of the study. The data will be processed and stored in compliance with data protection regulations.

**Wie alt sind Sie?** _________

**Which gender do you identify with?**

- Male
- Female
- Diverse
- Not specified

**What is your highest educational qualification?**

- Still a pupil
- Finished school without qualification
- Secondary school certificate (*Hauptschulabschluss*)
- Secondary school certificate (*Realschulabschluss*)
- Completion of a technical secondary school, etc. (*Fachhochschulreife*)
- Completed vocational training (*Berufsausbildung*)
- High school diploma (university entrance qualification)
- University degree
- Other educational qualification, namely: _____________________
- Not specified

**What is your mother tongue?**

*Several answers possible*

- German
- Other
- Not specified

**Thank you very much for your participation!**

Please hand in the questionnaire to the clinic staff. Please take the patient information sheet (first sheet) home with you and keep it in a safe place. You will find your personal identification number on the form. Please enter this number if you wish to view, block or delete your data. As we do not collect any contact details, it is not possible to process your request without the code.

Seite 4
