# Supplementary material for: Perceptions of Stigma Among Patients With Hepatitis B in Germany: Cross-Sectional Survey
Source: JMIR Form Res. 2025 Jun 13;9:e66379. doi: 10.2196/66379 (PMC12180674; doi:10.2196/66379)
Supplement: Multimedia Appendix 2 [file formative-v9-e66379-s002.docx]

**Additional file 1: Assumptions checks for regression analyses**

Cook’s distance indicated that the data contained no outliers (highest value = .068), suggesting no individual cases were unduly influencing the model. Collinearity statistics (Tolerance, VIF) indicated that multicollinearity was not a concern. Durbin-Watson test indicated that the data met the assumption of independent errors (Durbin-Watson = 2.144). Normal P-P plot of standardized residuals indicated that the data contained normally distributed errors (great majority of points lie on or very close to the line). Scatterplots showed that the relationship between the independent variables and the dependent variable is linear (relationships characterized by a straight line; note, two variables - gender and education - had to be dichotomized to meet this assumption); scatterplots of standardized predicted values showed that the data met the assumption of homoscedasticity (looking like a random array of dots). The data also met the assumption of non-zero variances.
